# Volitional Modulation of the Left DLPFC Neural Activity Based on a Pain Empathy Paradigm—A Potential Novel Therapeutic Target for Pain

**DOI:** 10.3389/fneur.2020.00714

**Published:** 2020-07-21

**Authors:** Carolina Travassos, Alexandre Sayal, Bruno Direito, João Castelhano, Miguel Castelo-Branco

**Affiliations:** ^1^Coimbra Institute for Biomedical Imaging and Translational Research (CIBIT), University of Coimbra, Coimbra, Portugal; ^2^Institute of Nuclear Sciences Applied to Health (ICNAS), University of Coimbra, Coimbra, Portugal; ^3^Siemens Healthineers, Lisbon, Portugal; ^4^Faculty of Medicine, University of Coimbra, Coimbra, Portugal

**Keywords:** neuroimaging, real-time fMRI, neurofeedback, pain empathy, dorsolateral prefrontal cortex

## Abstract

The ability to perceive and feel another person' pain as if it were one's own pain, e.g., pain empathy, is related to brain activity in the “pain-matrix” network. A non-core region of this network in Dorsolateral Prefrontal Cortex (DLPFC) has been suggested as a modulator of the attentional-cognitive dimensions of pain processing in the context of pain empathy. We conducted a neurofeedback experiment using real-time functional magnetic resonance imaging (rt-fMRI-NF) to investigate the association between activity in the left DLPFC (our neurofeedback target area) and the perspective assumed by the participant (“first-person”/“Self” or “third-person”/“Other” perspective of a pain-inducing stimulus), based on a customized pain empathy task. Our main goals were to assess the participants' ability to volitionally modulate activity in their own DLPFC through an imagery task of pain empathy and to investigate into which extent this ability depends on feedback. Our results demonstrate participants' ability to significantly modulate brain activity of the neurofeedback target area for the “first-person”/”Self” and “third-person”/”Other” perspectives. Results of both perspectives show that the participants were able to modulate (with statistical significance) the activity already in the first run of the session, in spite of being naïve to the task and even in the absence of feedback information. Moreover, they improved modulation throughout the session, particularly in the “Self” perspective. These results provide new insights on the role of DLPFC in pain and pain empathy mechanisms and validate the proposed protocol, paving the way for future interventional studies in clinical populations with empathic deficits.

## Introduction

Pain is one of the most used processes to invoke empathy. Imagining oneself (“first-person”/”Self” perspective) or another person (“third-person”/”Other” perspective) in painful situations encompasses a process of perspective taking which, in turn, is a stepping stone to human empathy—the capacity to share and understand the emotional states of others (1–4). The importance of this ability is highlighted in neuropsychiatric conditions characterized of empathic deficits, such as autism spectrum disorder (5, 6).

Neural networks involved in pain nociception are frequently triggered by the observation/imagination of pain (vicarious pain experience)—a process akin to classical synaesthesia (7–10). “Synaesthesia for pain” can be defined as mirror-sensory synaesthesia since brain regions that are activated during vicarious experience of pain seem to mimic pain nociception (8). The shared pain network between pain nociception and vicarious pain experience comprises parts of the pain matrix—the neural network responsible for experiencing pain (7–10). This network comprehends an affective-motivational (including anterior cingulate/midcingulate cortices—ACC/MCC, and anterior insula—AI) and a sensory-discriminative (including somatosensory cortices—S1 and S2) components. Although non-nociceptive pain stimuli can elicit similar cortical responses of being in physical pain, there are systematic differences in activation sites (9–14). The affective component of the pain matrix and the somatosensory cortices are usually activated when subjects are asked to view/imagine painful situations happening to themselves (“Self” perspective) (9–14). On the other hand, one's ability to take the perspective of others and understand their emotions (Theory of Mind, ToM) is strongly related with empathy for pain (15, 16). In this sense, the “Other” perspective, as an example of perspective taking, involves the activation of the precuneus, temporoparietal junction (TPJ), superior temporal sulcus (STS), middle temporal gyrus, middle frontal gyrus, and prefrontal cortex, a network of regions previously identified in ToM studies (9–14).

Previous studies have documented brain responses to vicarious painful experiences in the main brain regions of the putative pain matrix. However, recently, a systematic review of fMRI studies on empathy for observed pain identified a cluster on the left dorsolateral prefrontal cortex (DLPFC) (17). Several neuroimaging experiments involving attention, working memory, and goal-directed processes have highlighted the significance of this brain region (18). Additionally, it is also engaged in cognitive perspective taking or ToM, contributes to cognitive and emotional control processes, and is part of the circuitry of emotion regulation (2–4). In the context of pain experiments, its activation is expected to mediate part of the attentional-cognitive dimensions of pain processing associated with localization and encoding of the attended stimulus (19). Nevertheless, the role of the DLPFC in human empathy for pain is still a matter of debate.

We hypothesize that if the DLPFC is a critical region in the modulation of pain empathy then participants should be able to learn to volitionally control the activity in this region as a function of pain empathy imagery. This could potentially help improve one's ability to feel the pain of others, the defining condition of empathic disorders. To this end, we designed a neurofeedback experiment combining a real-time functional magnetic resonance imaging (rt-fMRI-NF) and a pain empathy task to measure activity in the left DLPFC in response to the imagery of painful experiences across “Self” and “Other” perspectives. The main goals of this study were 2-fold: (1) to investigate the subjects' ability to volitionally modulate the neural activity of the DLPFC through an imagery task of pain empathy, and investigate into which extent it is feedback-dependent; (2) to clarify the role of the DLPFC in a pain empathy paradigm that requires the imagination of painful situations while taking two different perspectives.

## Methods

### Participants

Seventeen healthy adult volunteers (8 female), aged between 23 and 32 years (mean age = 27 years, SD = 3.03), with no history of neurological or psychiatric diseases were included in this study. Participants gave written informed consent to take part in the study, which was approved by the local Ethics Commission (Faculty of Medicine of the University of Coimbra) and conducted in accordance with the Declaration of Helsinki.

### Experimental Protocol

**Figure 1:**
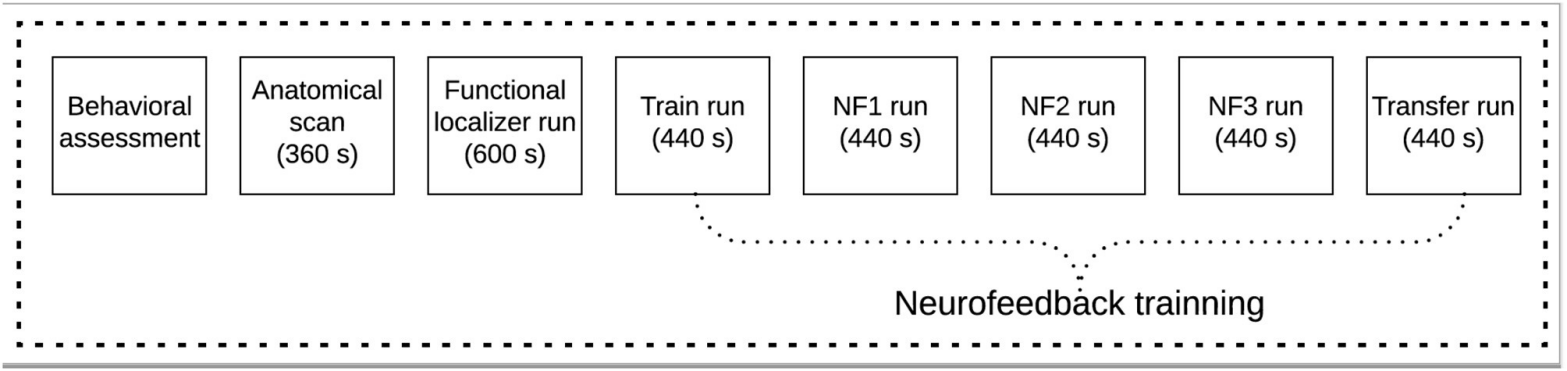
Experimental protocol scheme for the neurofeedback based on real-time functional magnetic resonance imaging (rt-fMRI-NF) pain empathy task.

Before the scanning session, each volunteer completed a behavioral assessment. Then, a scanning session consisting of one anatomical acquisition and six functional runs (one functional localizer, to select the target region of interest, and five imagery runs) were performed (Figure 1).

#### Behavioral Assessment

Participants completed a demographic information questionnaire and the Portuguese versions of two mood questionnaires: State-Trait Anxiety Inventory—State and Trait (STAI Y-1 and STAI Y-2) (20), and Davis' Interpersonal Reactivity Index (IRI) (21, 22).

STAI is a measure of the severity of the overall anxiety level that assesses both state (STAI Y-1) and trait (STAI Y-2) anxiety separately. Each type of anxiety scale has its own scale of 20 different questions that are scored from 20 to 80. Higher scores are positively correlated with higher anxiety levels. Answers are given using a four-point Likert scale, ranging from (1) not at all/almost never to (4) very much so/almost always, for state and trace anxiety, respectively. For the Portuguese population, the mean (and standard deviation) values for the STAI Y-1 questionnaire are, according to a previous study, 38.20 ± 10.70, 36.31 ± 9.61, for female and male cohorts, respectively; mean (and standard deviation) values for the STAI Y-2 are 37.30 ± 7.090, 34.98 ± 8.35 (20). These psychometric questionnaires are used to exclude potential confounding effects from personality traits or current mood states.

IRI is a 24-item self-report survey used as a measure of empathic abilities. This measure has 4 subscales, each made up of 6 different items. These subscales are (the subscale scores for the Portuguese population are also presented in the description, mean and standard deviation, female and male, respectively [9]): Perspective Taking—PT, reflect the tendency to spontaneously adopt the psychological point of view of others (2.71 ± 0.67, 2.48 ± 0.67); Fantasy—FS, denote a tendency of the respondent to transpose themselves imaginatively into the feelings and actions of fictitious characters in books, movies, and plays (2.25 ± 0.81, 2.04 ± 0.40); Empathic Concern—EC, assesses “other-oriented” feelings of sympathy and concern for others negative experiences (3.03 ± 0.62, 2.36 ±0.65); and Personal Distress—PD, measures “self-oriented” feelings of personal anxiety and unease when witnessing the negative experiences of others (1.83 ± 0.64, 1.68 ± 0.59). Since it is constructed as four independent measures ranging between 0 and 28 (each one with a unique psychological meaning) there is not a single composite score (21, 22).

We present the mean and standard error values across subjects for each scale and subscale.

#### Stimuli Characterization

**Figure 2:**
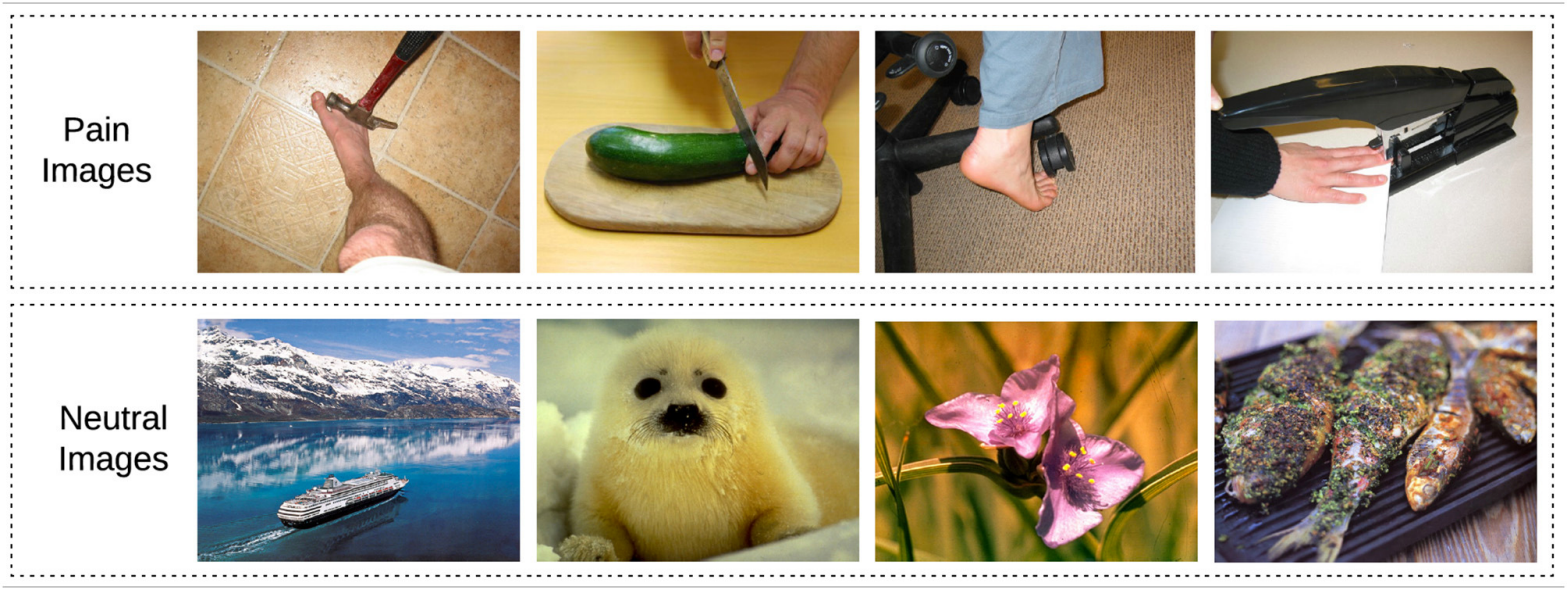
Picture stimuli used in the localizer and neurofeedback tasks. Top row: examples of painful situations that can occasionally happen in daily life (23, 24). Bottom row: example of neutral pictures (25).

Pain images used in the localizer and neurofeedback tasks were collected from the Visually-Induced Pain Empathy Repository (VIPER) (23) and Philip Jackson^1^ (24) databases. Various types of pain (mechanical, thermal, and pressure) representing potential painful situations that might occur in everyday life (such as cutting a finger with a knife—see Figure 2 for examples) were presented. Neutral images depicting neutral situations, objects, animals or plants (such as a book or a cupcake—see Figure 2 for examples) were selected from the International Affective Picture System (IAPS) database (25). To validate these stimuli, we asked an independent group of subjects (18 subjects) to rate the pictures regarding their arousal and valence content. Ratings were done using SAM scales (Arousal: from 0 = calm, to 8 = excited; Valence: from −4 = negative emotions, to 4 = positive emotions) (26). The data from these ratings was first normalized between 0 and 1, and the mean for each image across the 18 subjects was calculated. We then compared the ratings of pain and neutral images using a two-sample *t*-test.

#### Functional Localizer

**Figure 3:**
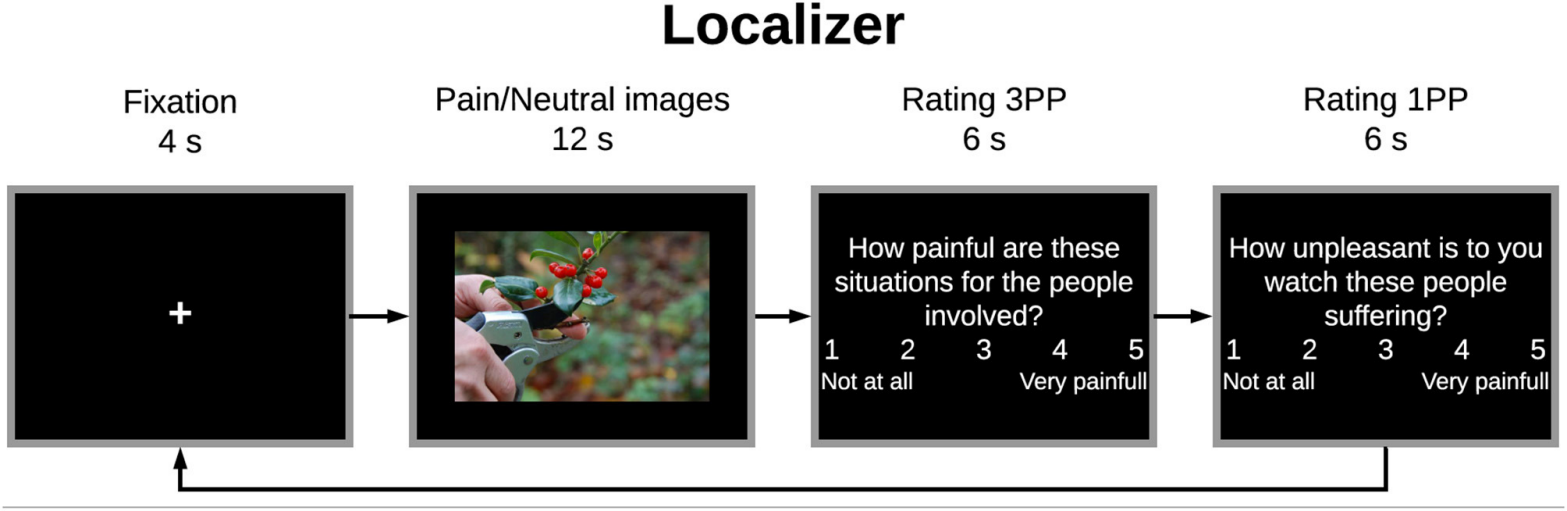
Protocol scheme for each trial of the localizer run: trial started with a fixation cross (4 s) followed by a set of 7 painful or neutral images (12 s)—alternated between trials, ending with two behavioral ratings regarding empathy for the pain of the other (rating “third-person” perspective−3PP) and personal distress (rating “first-person” perspective−1PP) (6 s per rating).

To functionally localize the neurofeedback target region—left DLPFC—of each subject, we adapted a picture-based paradigm developed by Yao et al. (27). A meta-analysis study of Lamm et al. (10) demonstrated that brain areas associated with inferring and representing mental states of “Self” and “Other”, namely the DLPFC, are frequently engaged in this type of experiments (instead of cue-based paradigms). To this end, a set of pictures depicting painful or neutral situations (7 images per trial) were presented alternately to the participants in a block design paradigm. Each trial started with a fixation cross (4 s) followed by a set of 7 neutral or painful images (12 s) and finished with two behavioral ratings of 6 s each (Figure 3). In total, the localizer is composed of 11 neutral-images trials alternating with 10 painful-images trials, each one lasting 28 s (an additional fixation cross was added at the beginning and the end of the run). The duration of this run was 10 min.

Participants were instructed to just watch each set of images and then answer the two behavioral ratings according to those images. The first question assessed other-oriented empathic response by judging the intensity of pain induced by the situations illustrated in the images (“How painful are these situations for the people involved?”); the second question assessed self-oriented distress via experienced unpleasantness (“How unpleasant is it for you to watch these people suffering?”) (28, 29). Participants had to rate using a 1–5 Numeric Visual Analog Scale (VAS): 1 indicating no pain/unpleasantness at all and 5 indicating the worst pain/unpleasantness. The aim of these ratings, beside ensuring the engagement of participants in the task, was to explore the relation between the two perspectives. The ratings were first normalized between 0 and 1 and then we estimated Pearson's correlation between the two.

#### Region-of-Interest (ROI) Definition

The functional localizer run allowed us to select a seed region-of-interest (ROI) in the left DLPFC of each participant to derive feedback signal to the neurofeedback training that followed. Each ROI was selected considering anatomical references (left frontal lobe covering the middle frontal gyrus) and the statistical activation map contrasting “Pain Images > Neutral Images” based on the online General Linear Model (GLM) produced by Turbo-BrainVoyager 3.2 software (Brain Innovation, Maastricht, The Netherlands). The target ROI was defined as the set of voxels surpassing the statistical threshold *t*-value = 3. The number of voxels was not constrained. This approach considers inter-subject variability and ensures an optimal selection of voxels for subsequent neurofeedback calculation.

#### Neurofeedback Training

**Figure 4:**
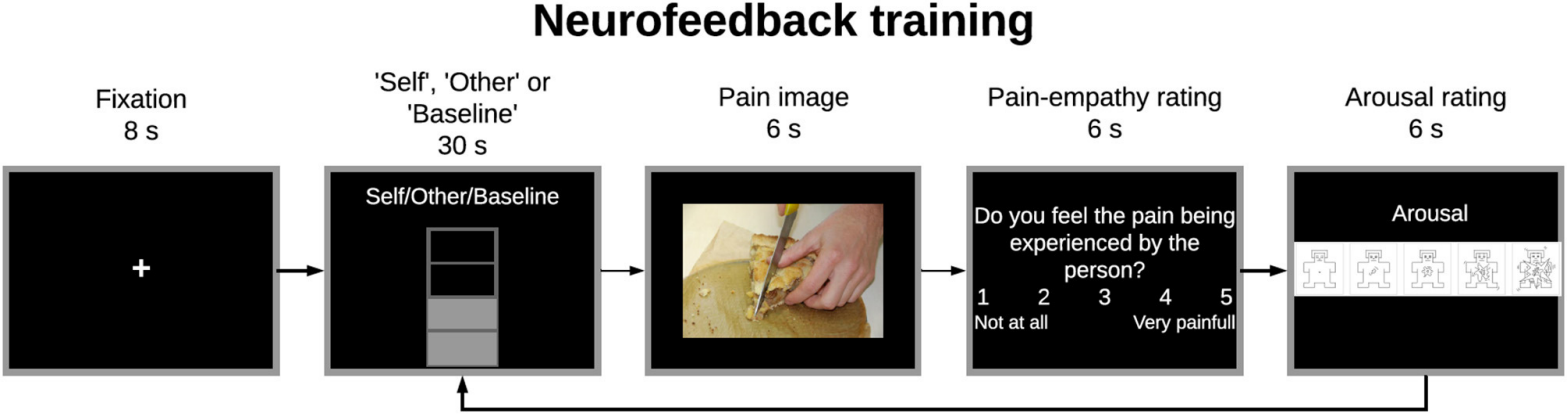
Experimental paradigm for the train, neurofeedback training, and transfer tasks. Participants had to imagine painful situations according to the “Self”/”Other” perspective presented at the begin of the imagery block to up-regulate the brain activity of the specified region-of-interest (ROI) (30 s). Then, participants viewed a potential painful picture (6 s) and answered two behavioral ratings concerning pain empathy and arousal (6 s per rating). In the train and transfer runs no feedback information was given during the imagery block.

The neurofeedback training comprised one pre-training run (train), three training runs (NF1, NF2, NF3), and one post-training run (transfer), each one with 9 trials. Up- and down-regulation trials were alternately presented to the participants. In both cases, subjects had 30 s to imagine painful or pleasurable situations according to the perspective presented and then, 6 s to view a potential painful picture and 12 s to answer two behavioral ratings (Figure 4). Therefore, each trial had 48 s and each run lasted about 440 s (a fixation cross of 8 s was added at the beginning of each run).

In the up-regulation trials, participants had to assume the “Self” or “Other” perspective showed at the top of the feedback-thermometer and imagine themselves or a loved-one (respectively) in painful situations to increase the number of filled bars of the thermometer (which means higher activity when compared to the baseline period); in the down-regulation trials, they were instructed to return the thermometer values to the baseline level (decrease the number of filled bars) by imaging pleasure situations. Before the experiment, participants were informed that they should not recall previous situations to prevent mixing with working memory load. Regarding the behavioral ratings, in the first question participants had to assess in a 1–5 VAS how much they felt the pain being experienced by the person in the picture (from 1 = not at all, to 5 = worst pain) (pain empathy rating) and, in the second question, the arousal of the presented image using the Self-Assessment Manikin (SAM) scale (from 1 = calm to 9 = excited) (26). The aim of these ratings, beside ensuring the engagement of participants in the task, was to explore the relation between the two perspectives. The ratings were first normalized between 0 and 1 and then we estimated Pearson's correlation between the two.

#### Feedback Calculation

The BOLD signal of the target ROI was extracted, and the feedback signal was discretized in 10 intervals and translated into a visual representation (thermometer) to present in real-time to subjects.

The feedback calculation was based on the ratio between the blood oxygenation dependent level (BOLD) signal values during the up- and down-regulation blocks:

$$\begin{array}{l} SignalVar\left(\%\right)=\frac{x\left(t\right)-b\left(t\right)}{b\left(t\right)}\;\times \;\frac{100}{maxPSC} \end{array}$$

where x(t) is the BOLD value at time point t, b(t) is the baseline value for the same time point t (calculated based on the down-regulation condition prior to the active modulation condition), and maxPSC is a threshold that defines the maximum percent signal value (set to 2.5 based on the pilot acquisitions previous to the protocol definition). Due to the BOLD response delay, the values defined by the baseline interval should consider the hemodynamic delay. To this end, the first 3 volumes of the down-regulation block were excluded, and the first volume of the next block was included in the calculation of the baseline condition values. The feedback information was a percent signal value used to fill the thermometer display presented in real-time.

The participants were informed of the hemodynamic delay (~6 s). This type of feedback has the advantage that participants can adaptively test different mental strategies to optimize the modulation of the target brain area. On the first and last runs (train and transfer) no feedback was given.

### fMRI Data Acquisition

MRI data were acquired on a 3 Tesla (T) Siemens Magnetom Tim Trio scanner (Siemens, Erlangen, Germany) equipped with a 16-channel head coil, at the Institute of Nuclear Sciences Applied to Health (ICNAS), Coimbra, Portugal. A T1-weighted Magnetization-Prepared Rapid Gradient-Echo (MPRAGE) sequence was used to obtain the anatomical image [matrix size: 256 × 256; 176 slices; 1 mm^3^ isotropic voxels; repetition time (TR): 2,530 ms; echo time (TE): 3.42 ms; field of view (FoV): 256 mm; flip angle (FA): 7°]. After the structural image sequence, six functional runs were acquired. Changes in the BOLD T2^*^-weighted MR signal were measured using a gradient echo-planar imaging (EPI) pulse sequence of 32 slices (TR: 2,000 ms; TE: 3.0 ms; FoV: 210 mm; matrix size: 70 × 70; in-plane resolution: 3 × 3 mm^2^; slice thickness: 3 mm; slice gap = 0 mm, FA: 75°). A total of 300 volumes were acquired for the first functional run and 220 volumes for the following runs. The session duration was ~1 h.

### fMRI Data Analysis

#### Online Analysis

The presentation of the feedback signal in real-time was performed using Turbo BrainVoyager v3.2 (Brain Innovation, Maastricht, The Netherlands). The data acquired during the functional runs were pre-processed using slice scan time correction, 3D motion correction and linear trend removal.

#### Offline Analysis

Offline data analyses were performed using BrainVoyager v21.0 (Brain Innovation, Maastricht, The Netherlands). Pre-processing of single-subject functional data included slice-time correction, 3D motion correction aligning to the first functional volume, and temporal high-pass filtering (GLM-Fourier with 2 sin/cos) to remove low-frequency drifts. Functional and anatomical data were then co-registered and normalized to Talairach coordinate space (30). A 6-mm full-width-half-maximum Gaussian kernel was used to spatially smooth the data.

Regarding statistical analysis of the neuroimaging data, activation maps were computed using the GLM. The first-level design matrix included predictors encoding the experimental conditions, six motion parameters (three translational and three rotational) and spikes detected (based on the root mean square displacement between volumes). Second-level, group analyses were performed using Random Effects (RFX) analysis to allow for population inferences. All statistical maps were corrected for multiple comparisons using False Discovery Rate [FDR, q(FDR) = 0.03] method. To prevent repetition errors and increase processing times, these analyses were automated with MATLAB R2018a (Mathworks, Inc., Sherbon, MA, USA) and the COM functionality of BrainVoyager (Brain Innovation, Maastricht, The Netherlands).

#### Target ROI Analysis

In order to assess the ability of each subject to modulate the target region, a ROI-GLM statistical analysis was performed considering the target ROI of each participant. We extracted the beta values of “self”, “other”, and “down-regulation” for (i) train, (ii) neurofeedback (3 runs) and (iii) transfer runs; and estimated the statistical significance of the contrasts Self > Down-regulation, Other > Down-regulation, and Self > Other based on two-tailed paired *t*-tests.

## Results

### Behavioral Assessment

Participants that took part in our study reported an average of 63.88 ± 11.98 on STAI Y (STAI Y-1: 30.47 ± 6.24; STAI Y-2: 33.41 ± 7.01). Regarding the IRI scale, participants reported an average of 17.71 ± 3.43, 12.00 ± 4.23, 16.53 ± 3.85, and 10.06 ± 3.47 on PT, FS, EC, and PD subscales, respectively.

### Stimuli Characterization

**Figure 5:**
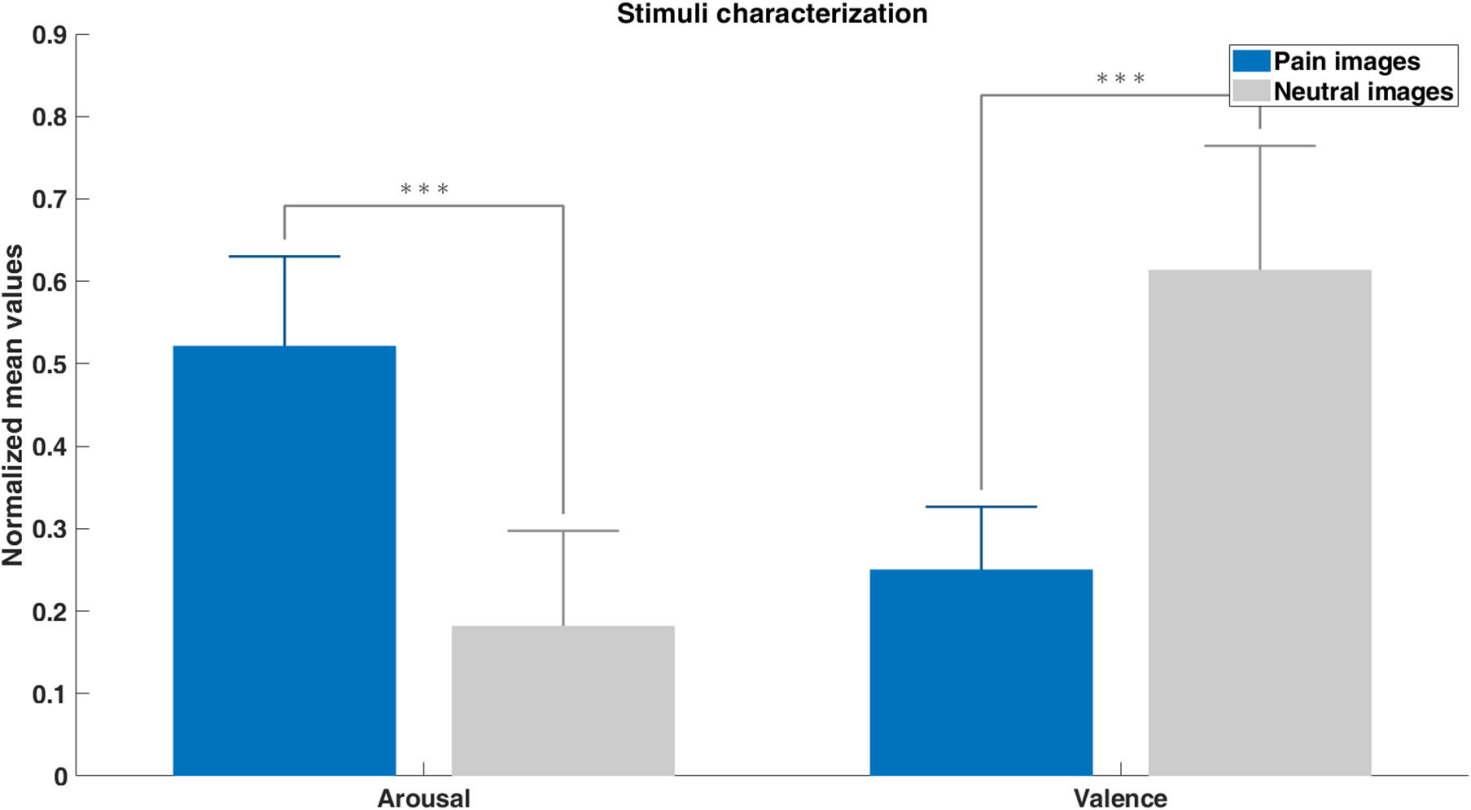
Pain and neutral images characterization based on the arousal and valence ratings given by an independent group of subjects (feature scaled mean values + SE). Differences between pain and neutral stimuli were assessed with unpaired *t*-tests (***p < 0.001).

The localizer and neurofeedback tasks included arousing pain pictures and neutral pictures previously validated by an independent group of subjects. Average of arousal and valence ratings were feature scaled and represented in Figure 5. Arousal ratings showed that subjects rated the painful stimuli with significantly higher arousal values than the neutral ones. Lower valence ratings for pain images reflects their negative content. These ratings validate the difference in the content of the pain and neutral images.

### Online Definition of the Target ROI (Left DLPFC)

**Figure 6:**
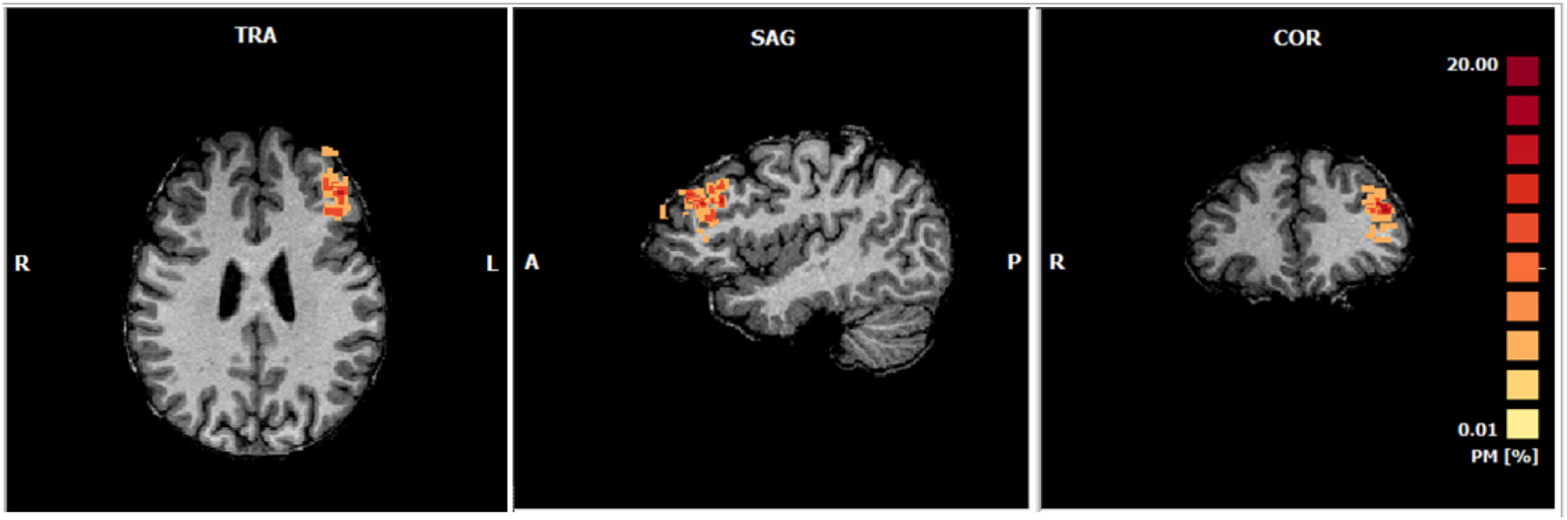
Probabilistic maps of the ROIs selected online during the functional localizer of each subject (Talairach center of gravity mean coordinates: −39.18 ± 2.93, 35.09 ± 7.66, 27.85 ±7.53; Number of voxels: 393). The percentage of subjects in which each voxel was selected is represented accordingly to the color range from yellow to red.

Figure 6 shows the probability map of ROIs positions which represents the spatial consistency of online selection of the target region across subjects. The mean ROI size in 1 mm resolution is 393 ± 52 voxels and a complete description of the selected ROIs for each subject is presented in Supplementary Table 1.

### Offline Identification of Brain Regions Involved in Pain Representation

**Figure 7:**
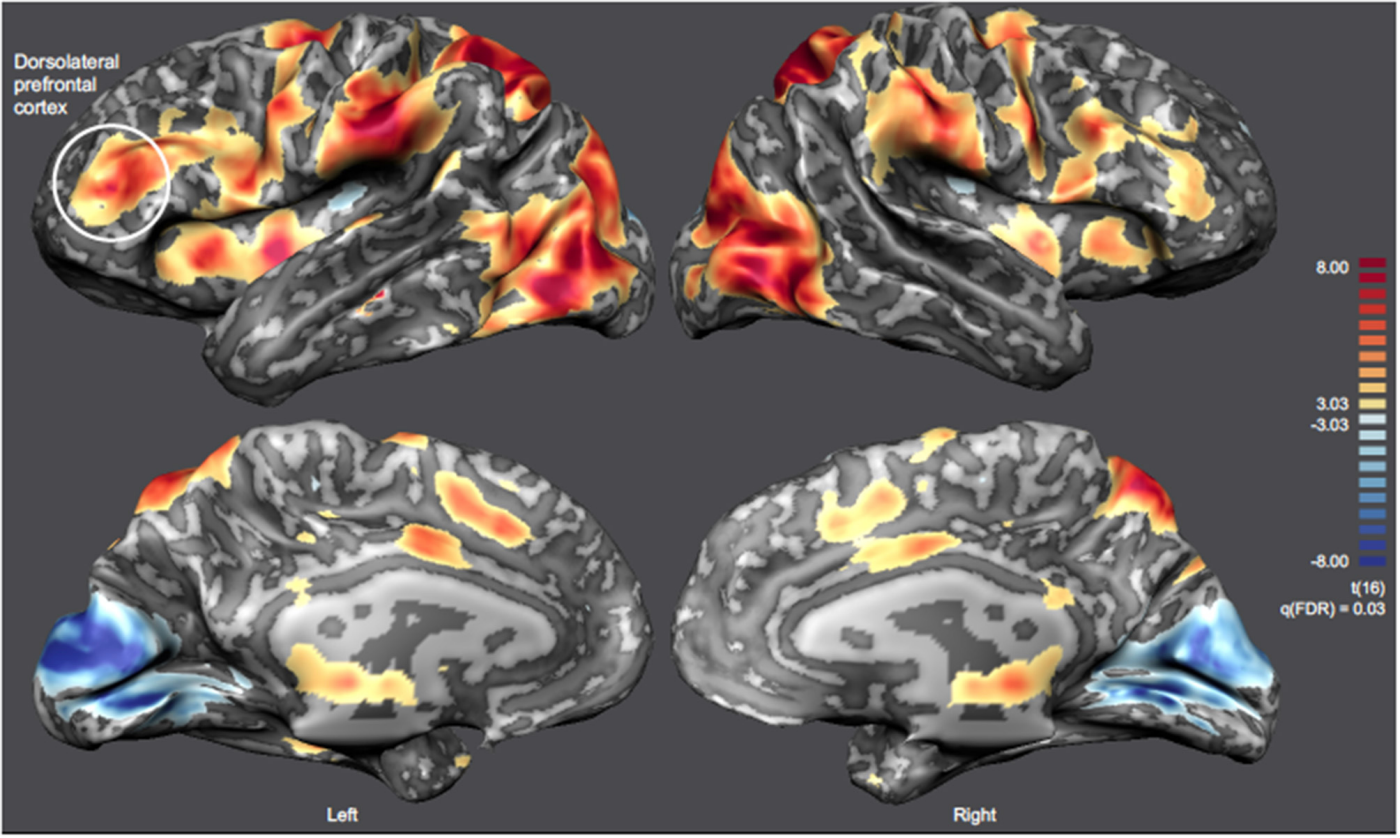
Inflated cortex representation of the statistical map corresponding to the contrast Pain Images > Neutral Images [RFX-GLM, q(FDR) = 0.03] of the localizer run. The neurofeedback target (left DLPFC) is highlighted and the remaining clusters are summarized on Supplementary Table 2.

The offline analysis of the whole-brain group activation map of the functional localizer (Figure 7) considering the contrast Pain Images > Neutral Images, allowed us to identify the network involved in pain perception and processing, including the left DLPFC [RFX, q(FDR) = 0.03]. Statistically significant clusters were identified in several brain regions related to pain processing, such as the ACC and the AI (complete description of these regions is presented in Supplementary Table 2). A consistent group activation of the left DLPFC (highlighted in Figure 7) is also evident. The ROI on the left DLPFC align with those previously defined online during the localizer task (see Supplementary Figure 1).

### Imagery Runs Analysis—ROI-GLM Approach

**Figure 8:**
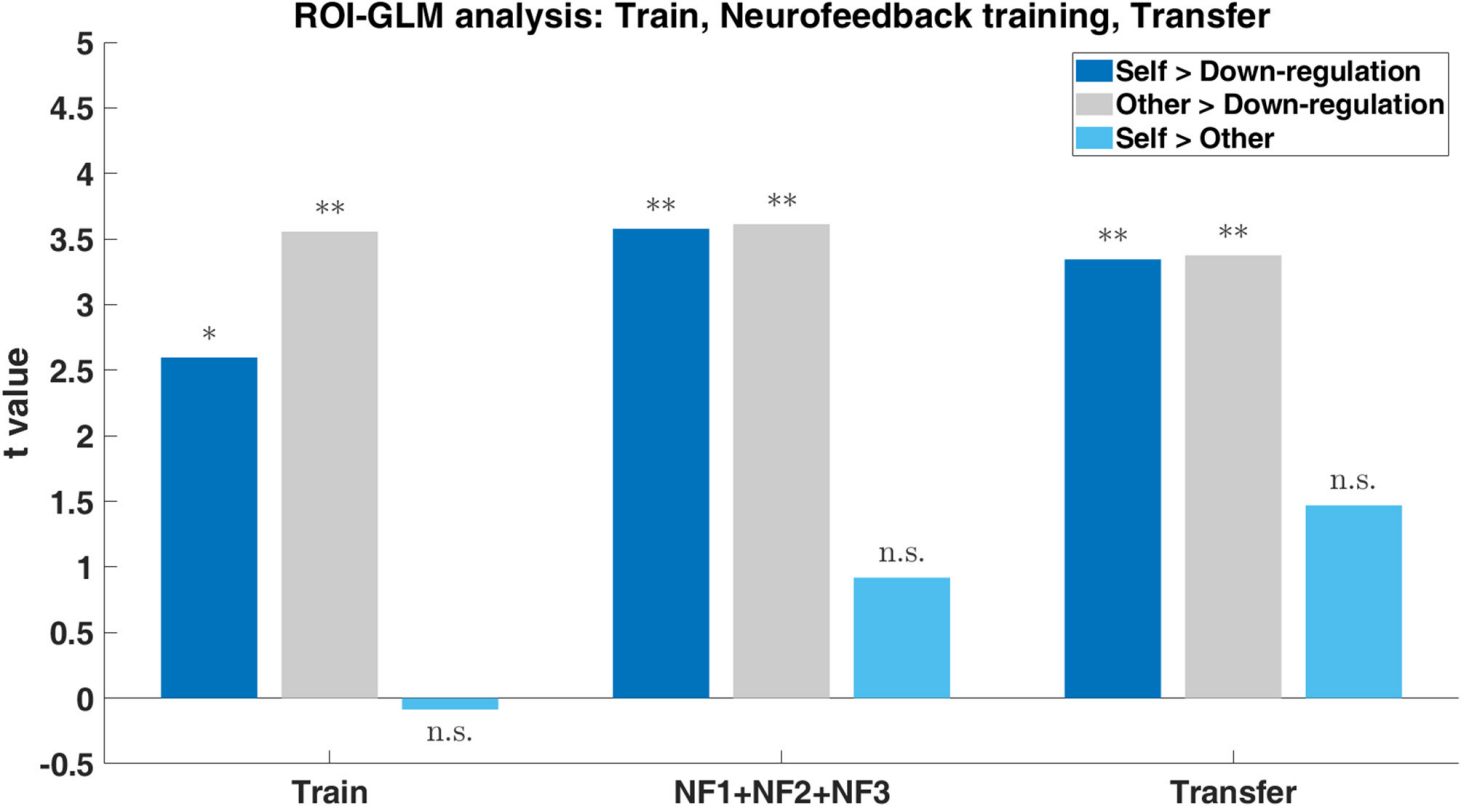
Group results (*n* = 17) to the three imagery tasks (“Self”: imagine itself in painful situations; “Other”: imagine a loved-one in painful situations; “Down-regulation”: imagine pleasure situations) within the ROI on the left-DLPFC during the train, neurofeedback training (NF1, NF2, and NF3) and transfer. Bar plot of the *t*-values for the contrasts Self > Down-regulation (dark blue), Other > Down-regulation (gray), and Self > Other (light blue) with *p*-values significance (*0.05, **0.01).

ROI-GLM statistical analysis of the experimental runs highlighted the subjects' ability to modulate the brain activity of the neurofeedback target (ROI on the left-DLPFC) with both “Self” and “Other” perspectives (when subjects were asked to imagine themselves or a loved-one in painful situations, with the goal of increasing the ROI activity; contrasts: Self > Down-regulation and Other > Down-regulation, respectively)—Figure 8. Both perspectives present statistically significant modulation of the left DLPFC already in the train run. The “Other” perspective presents statistically significant results throughout the session, maintaining the modulation. The “Self” perspective also sustains successful modulation and even shows a trend for improvements throughout the session. Results regarding the direct comparison between imagine itself or a loved-one in painful situations (contrast: Self > Other) reveal no statistically significant differences in the ROI analysis of any run (Figure 8). This shows that neuromodulation can be achieved regardless of “Self” vs. “Other” strategies. Detailed results are presented in Supplementary Table 3.

### Imagery Runs Analysis—Whole-Brain Approach

**Figure 9:**
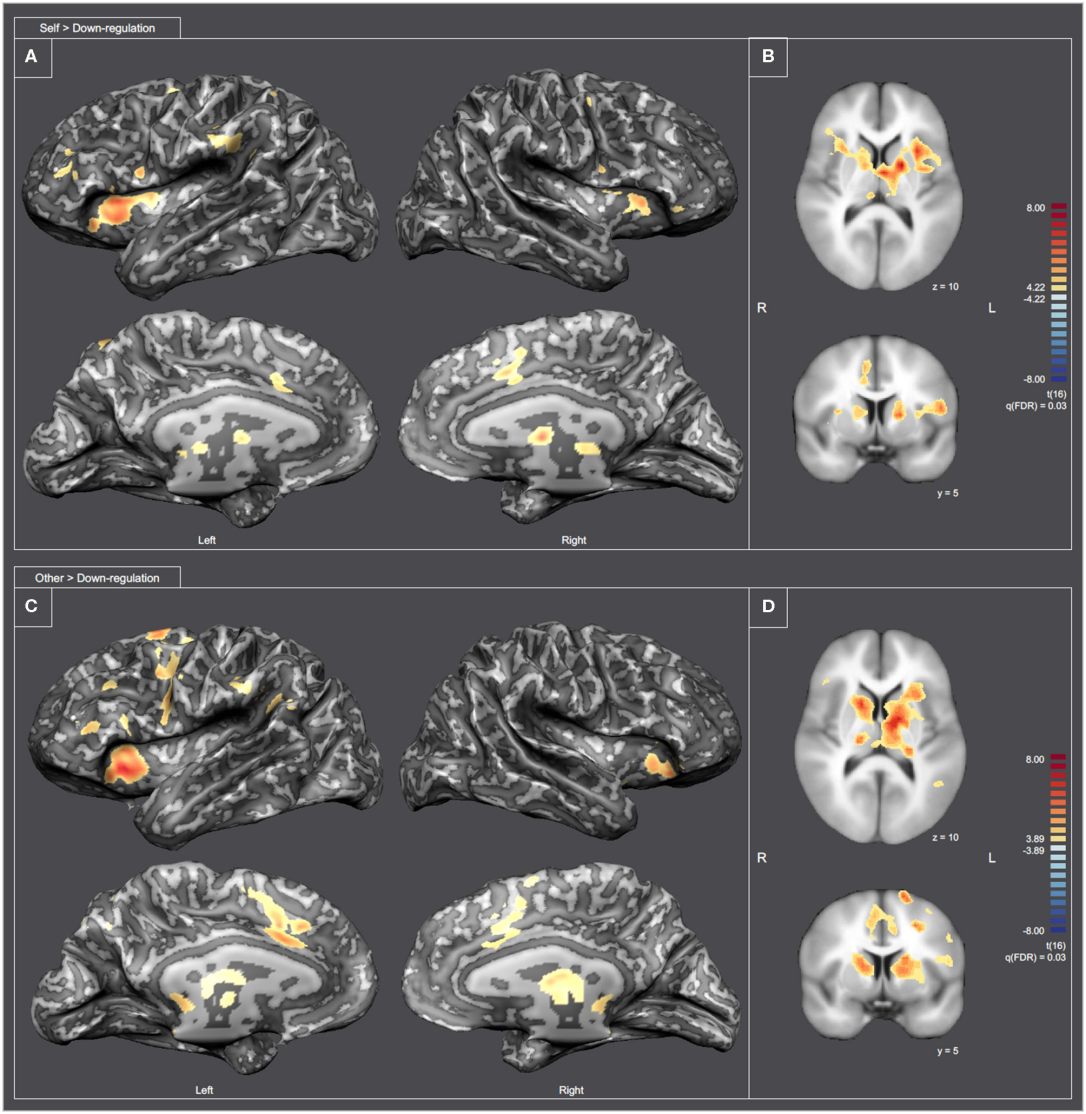
Statistical map representation for the imagery runs [RFX-GLM, q(FDR) = 0.03]. **(A,C)** Display inflated cortex representations for the contrasts Self > Down-regulation and Other > Down-regulation, respectively. **(B,D)** Display subcortical activations projected in axial (Z = 10) and coronal (Y = 5) slices for the contrasts Self > Down-regulation and Other > Down-regulation, respectively.

To better understand the differences between the “Self and Other” perspectives at the whole brain level, we analyzed the contrasts Self > Down-regulation and Other > Down-regulation [RFX, q(FDR) = 0.03] (including all experimental runs and all subjects). Figure 9 combines inflated cortex representations of cortical activations (9A and 9C), and representation of subcortical activations projected in axial (Z = 10) and coronal (Y = 5) slices (9B and 9D) of contrasts of interest (Self > Down-regulation and Other > Down-regulation, respectively). The main clusters were also selected and labeled—Supplementary Tables 4, 5, respectively. Both perspectives were associated with activations in the neural network involved in pain processing, including the bilateral insula, cingulate cortex, and the thalamus. However, the activation of the somatosensory cortex and intraparietal sulcus (IPS) are specific of the “Self” perspective. Conversely, activation of the TPJ, supramarginal gyrus, and precuneus are specific of the “Other” perspective. Both perspectives engaged the premotor cortex and the reward network very extensively, including different structures of the dorsal striatum, namely left putamen and right caudate. The statistical activation map suggests that ventral striatum is not involved.

### Correlation Analysis of Behavioral Online Ratings

We also asked participants to provide two behavioral ratings during the functional scanning runs. The first rating assessed other-oriented empathic response (regarding the pain in the other or pain empathy) and the second-one the pain-related discomfort (regarding the self-unpleasant or arousal). Based on the behavioral ratings of the localizer run (“How painful are these situations for the people involved?”; “How unpleasant is it for you to watch these people suffering?”), we found that ratings for pain in others (related to the first question) were positively correlated with ratings for self-unpleasantness (second question) (Table 1). Similarly, the relationship between ratings corresponding to pain empathy (“How much do you feel the pain being experienced by the person in the picture?”) and arousal (“How did that image make you feel?”) during the neuromodulation part (independent of the assumed perspective), was significant (Table 1).

**Table 1:** Correlation analysis of behavioral ratings of the localizer and the neurofeedback runs (independent of the assumed perspective) (r—correlation coefficient; p—statistical significance).

		***r***	***p***
Localizer	Pain in Others vs. Self Unpleasant	0.772	*p* < 0.01
Train	Pain empathy vs. Arousal	0.861	
NF1		0.738	
NF2		0.660	
NF3		0.628	
Transfer		0.701	

## Discussion

Our study combined a rt-fMRI-NF pain empathy imagery-based paradigm with a perspective taking component to assess volitional control of the left DLPFC activity in healthy subjects. Previous studies showed that rt-fMRI-NF can be used as a tool for learning how to modulate the activity of localized brain regions (31). However, there is a gap in the current literature regarding rt-fMRI-NF studies that focus on pain empathy. Furthermore, the role of the DLPFC in the context of pain empathy experiments involving a perspective taking component has also not been studied. Our study aimed to fill this gap using an imagery paradigm of painful scenarios from two different perspectives (“first-person”/“Self” and “third-person”/“Other” perspective), as a tool for rt-fMRI-NF targeting the left DLPFC.

The functional localizer task allowed us to select an individual ROI on the left DLPFC of each subject (based on the contrast Pain Images > Neutral Images) to give real-time feedback information in the following neurofeedback runs. The overlap between the regions of interest selected on-line demonstrates that the approach allows a consistent identification of the target region. In the whole-brain group activation map of the localizer task, besides the cluster corresponding to the target region, we also observed the pattern of activation consistently found in previous pain empathy studies (7–10). Core regions involved in pain representation and processing (such as the insula and cingulate cortex, which are at the core of the salience network) were identified.

To assess the success of the neuromodulation strategies, we performed a ROI(target region)-GLM analysis of the experimental runs. We found that participants were able to modulate the brain activity of the target regions with both perspectives throughout the session. In particular, the participants were able to modulate (with statistical significance) the activity already in the first run of the session (i.e., train run) for both perspectives even without training and feedback information. The surprising ability to neuromodulate early on explains why no room for improvement was needed for the “Other” perspective and only a trend for the “Self” perspective. Taken together, these results demonstrate that both strategies (different perspectives in pain/pain empathy imagery) are appropriate to successfully modulate the left DLPFC. Additionally, we also investigated the differences between perspectives (contrast: Self > Other). The results showed no statistically significant differences in the ROI analysis of any run, which is also consistent with the behavioral data. Considering the results of the ROI-GLM analysis, we hypothesize that functional modulation of this region, which receives input from regions from the salience network (such as the insula), allows to provide cognitive weighting to information stemming from this network, shift the focus of attention, hold pain information in working memory, and potentially access the motor system if readiness for escape or defense responses would be necessary. In fact, there are neuroimaging and neuromodulation pain studies that highlight these features of DLPFC (14, 27, 32, 33). These results add to the understanding of the functional relevance of the left DLPFC in the modulation of empathy for pain, which has been a less studied subject in the field of pain-empathy.

Regarding the whole-brain analysis of the experimental runs, we expected that the mental simulation of pain in both perspectives tapped into the neural mechanisms of pain processing. Observing or imagining someone else in pain requires a perspective-taking procedure that produces a shared emotional experience. Therefore, modulation of attention toward others' experienced pain activates areas similar to when subjects orient toward the intensity of own felt pain. This mechanism is validated by the “shared-representations theory.” As described in the related literature, there is an overlap between the neural circuits involved in the experience of pain (or other emotions/sensations) and the observation/imagination of the same emotion/sensation (1, 2, 8–10, 13). In fact, our results point out to a partial overlap in the neural patterns of the imagination of pain in both perspectives with the main brain region of the pain matrix, which reflects analogous stimulus processing. Both perspectives engaged the main regions of the putative pain-matrix, namely the ACC, AI, and thalamus. However, some differences were expected between the neural patterns corresponding to each perspective (9–14). Based on previous literature, we expected stronger activation in the main components of the pain-matrix, namely on the ACC, AI, and S1/S2, when subjects took the “Self” perspective (which is closer to the situation of self-pain) (13). In contrast, taking the “Other” perspective would also lead to activation in prefrontal areas, namely in the DLPFC, since it is related to cognitive empathy and mental perspective taking (12, 16). The precuneus, TPJ, STS, and middle temporal/frontal gyrus were also expected to be activated in terms of ToM accounts (10, 13, 16, 34). Indeed, our results showed that the “Self” perspective engaged the pain matrix more extensively, as evidenced by the activity on the AI, ACC, thalamus and somatosensory cortex. The identified activation in the IPS also corroborates this engagement and is consistent with connections of this brain region with the ACC. The egocentric point-of-view of this perspective can explain the wide-ranging involvement of these brain regions. On the opposite, the precuneus, TPJ, and supramarginal gyrus were engaged exclusively in the “Other” perspective. These regions are involved in perspective taking, ToM, and play a crucial role in the sense of agency and self-identification (35). The involvement of the premotor cortex in both perspectives can be explained due to sensory-motor processes elicited by this type of paradigm (pictures of hands/feet in painful situations), including internal motor mimicry programs for potential avoidance (10). Yet, the similarities and differences between imaging the “Self” and the “Other” in pain are a key result of our study and their agreement with previous literature further validates our paradigm.

Lateralization in empathy-related processes was previously addressed in other studies (17, 36, 37), without a clear pattern. Bilateral activation of the AI has been previously reported in several tasks as others' pain imagery, watching facial expressions of pain, or observing static pictures of potentially painful situations (36). On the opposite, there are also reports indicating that the right AI was preferentially activated when subjects were asked to adopt the other's perspective (13). Interestingly, our data suggests a stronger recruitment of the left insula for both “Self” and “Other” perspectives. Both left and right DLPFC have been associated with pain-related processes. Previous studies suggested that different emotional regulation strategies are associated with a lateralized DLPFC activity: the left side is often involved with the meaning reinterpretation of the affective response, while the right side seems to play a role on psychological distancing from the emotional stimulus (37). Fallon and colleagues also explored the network involved in empathy for observed pain (17). The authors concluded on the specificity and lateralization of DLPFC regarding empathy for pain and direct experience of pain (left and right, respectively). Our results support the previous findings regarding the lateralization of empathy-related processes. The analysis of subcortical regions in the statistical maps revealed asymmetries between hemispheres. Our data suggests a stronger involvement of the right caudate and left putamen for both contrasts of interest. The involvement of the ventral striatum has been previously reported (38), however we found no significant activation of this region.

On the behavioral level, we asked participants to rate the pain/neutral images concerning the pain that would be caused in the other and the unpleasantness/arousal in oneself. The main goal of these ratings was to explore the emotional aspects of pain and confirm participants' engagement in the task. According to the literature, visualizing/imagining another person in pain elicits a series of manifestations including distress, anxiety, and discomfort (38). Our results suggest that when participants were instructed to mentalize the pain of the other or quantify empathy for the pain of the other, they are also in personal distress. These results provide evidence for the theory of “affective sharing” (emotional responses to others' emotions) (3).

Our goal with this study was to explore the self-modulation potential of a novel combination of pain-empathy imagery and the target region in healthy participants. For this reason, there are some limitations or issues that should be addressed in future studies. Regarding the selection of optimal control strategies for neurofeedback experiments, a very actual matter of debate (39), future clinical application in the area of pain research should help to identify the best clinical control group for the target application. In the design of an interventional study should also be considered that neuromodulation was successful even in the absence of feedback. Then, although our single-session experiment allowed us to identify immediate neuromodulation effects, estimation of effects in multiple-session interventional studies remains to be determined. Finally, the reconfiguration of the interactions within the neural network involved should also be considered. In this sense, an important follow-up would be to analyze the potential alteration of the functional connectivity patterns in the pain matrix. Despite these issues to be further investigated, the results from this proof-of-concept, feasibility study provide further indications that self-modulation of a key node of the pain matrix is possible. The ability of the participants to modulate activity allow us to conclude that the approach is feasible and has potential clinical implications. Further studies, with additional number of sessions and optimal control strategies, are necessary to clinically validate the neurotherapeutic potential of fMRI-aided self-regulation on pain/pain empathy. Understanding the role of the DLPFC in the context of empathy is of utmost importance, since it can be a potential interventional target in clinical studies of a wide range of clinical health issues characterized of empathic deficits. Moreover, its cortical location is suited for other neuromodulation techniques like Transcranial Magnetic Stimulation or Transcranial Direct Current Stimulation. Therefore, future work should focus on controlled/clinical-trial studies in clinical populations for whom the function of the DLPFC is compromised or that have deficits related to affective sharing.

## Conclusions

Taking the others' cognitive perspective and imagining their pain is crucial to our capacity to empathize. This study revealed the subjects' ability to modulate brain activity of the left DLPFC with a pain empathy neurofeedback paradigm involving “Self” and “Other” perspectives. Surprisingly, the ability to neuromodulate was achieved early on, even in the absence of prior training. Subjects did not even need feedback to improve the already established significant neuromodulation. Indeed, our results emphasized the importance of the proposed strategy for the subjects' ability to imagine the pain of others and modulate the region-of-interest brain activity accordingly. Overall, our results highlight the potential of the left DLPFC as a neuromodulation target in pain empathy experiments and encourage us to generalize this paradigm to a clinical population.

## Data Availability Statement

The raw data supporting the conclusions of this article will be made available by the authors, without undue reservation.

## Ethics Statement

The studies involving human participants were reviewed and approved by Ethics Committee of the Faculty of Medicine of the University of Coimbra. The patients/participants provided their written informed consent to participate in this study.

## Author Contributions

CT, AS, BD, JC, and MC-B conceived and designed the study. CT performed the recruitment and wrote the manuscript. CT and AS acquired and analyzed the data. CT, AS, BD, and MC-B discussed the results. AS, BD, JC, and MC-B reviewed the manuscript. All authors read and approved the final manuscript.

## Conflict of Interest

The authors declare that the research was conducted in the absence of any commercial or financial relationships that could be construed as a potential conflict of interest.

## Footnotes

^1^The picture stimuli were used with permission of Jackson et al. (24).
