# Supplementary material for: Volitional Modulation of the Left DLPFC Neural Activity Based on a Pain Empathy Paradigm—A Potential Novel Therapeutic Target for Pain
Source: Front Neurol. 2020 Jul 21;11:714. doi: 10.3389/fneur.2020.00714 (PMC7394699; doi:10.3389/fneur.2020.00714)
Supplement: Supplementary file 1 [file Table_1.DOCX]

**Supplementary Material**

**Table 1.** Properties of the regions-of-interest (ROIs) selected online during the localizer task for each subject based on the contrast Pain Images > Neutral Images. The size of the ROIs was according to the default threshold (3.0) in Turbo-BrainVoyager 3.2 (TBV). Cluster peak and center of gravity coordinates are in Talairach coordinates.

| ***Participant*** | ***Cluster peak coordinates*** | | | ***Center of gravity coordinates*** | | | ***Cluster size***  ***(voxels)*** |
| --- | --- | --- | --- | --- | --- | --- | --- |
|  | ***Peak x*** | ***Peak y*** | ***Peak z*** | ***Mean x ±SD*** | ***Mean y ±SD*** | ***Mean z ±SD*** |  |
| S01 | -32 | 30 | 38 | -35.70±2.44 | 27.78±3.24 | 39.21±2.43 | 513 |
| S02 | -35 | 27 | 32 | -40.21±3.21 | 26.00±1.91 | 30.64±1.78 | 317 |
| S03 | -39 | 43 | 34 | -38.26±2.34 | 40.63±1.83 | 33.51±0.88 | 136 |
| S04 | -43 | 35 | 26 | -44.30±2.89 | 29.75±2.31 | 23.38±1.71 | 415 |
| S05 | -35 | 27 | 32 | -35.60±2.39 | 27.60±2.39 | 31.00±0.82 | 135 |
| S06 | -38 | 38 | 29 | -36.36±2.27 | 41.57±2.74 | 28.05±2.28 | 379 |
| S07 | -44 | 29 | 24 | -43.41±3.53 | 32.28±2.44 | 18.88±2.52 | 429 |
| S08 | -32 | 30 | 38 | -35.73±2.33 | 29.11±2.50 | 37.51±1.63 | 324 |
| S09 | -33 | 30 | 38 | -36.10±2.36 | 57.83±3.31 | 24.93±1.89 | 410 |
| S10 | -38 | 35 | 12 | -42.00±2.30 | 33.23±1.48 | 11.38±1.00 | 117 |
| S11 | -37 | 38 | 16 | -35.03±2.26 | 38.77±2.40 | 13.14±1.95 | 366 |
| S12 | -41 | 36 | 29 | -40.60±3.58 | 28.57±3.81 | 29.09±2.29 | 641 |
| S13 | -41 | 36 | 29 | -39.35±3.00 | 31.67±3.02 | 32.46±1.92 | 259 |
| S14 | -41 | 36 | 29 | -42.06±3.04 | 39.54±3.15 | 27.86±2.40 | 551 |
| S15 | -39 | 43 | 34 | -42.12±2.86 | 42.16±2.63 | 33.70±1.70 | 380 |
| S16 | -41 | 36 | 29 | -40.26±3.71 | 33.89±2.83 | 25.30±2.57 | 1008 |
| S17 | -39 | 43 | 34 | -38.98±2.97 | 36.22±2.63 | 33.39±2.16 | 296 |

**Table 2**. Summary of the regions identified for the group localizer data, contrasting Pain Images > Neutral Images (RFX-GLM). We present, for each ROI peak voxel, the location using Talairach coordinates and the t-value for the contrast.

| ***Region*** | ***Brodmann Area*** | ***L/R*** | ***Peak voxel coordinates*** | | | ***t-Score*** |
| --- | --- | --- | --- | --- | --- | --- |
|  |  |  | ***X*** | ***Y*** | ***Z*** |  |
| Intraparietal Sulcus | 7 | R | 27 | -58 | 49 | 15.79 |
| Premotor cortex | 6 | R | 54 | 5 | 31 | 8.56 |
| Insula | 13 | R | 39 | -14 | -5 | 8.00 |
| Premotor cortex | 6 | R | 27 | -13 | 49 | 9.21 |
| Visual cortex | 17 | L | -6 | -76 | 7 | -11.46 |
| Amygdala |  |  | 15 | -13 | -2 | 7.72 |
| Cingulate cortex | 24 | L | 0 | -4 | 37 | 6.72 |
| Supramarginal gyrus | 40 | L | -54 | -25 | 34 | 15.02 |
| Premotor cortex | 6 | L | -27 | -10 | 55 | 9.88 |
| Insula | 13 | L | -33 | 14 | 4 | 7.44 |
| Insula | 13 | L | -36 | -4 | 1 | 10.92 |
| Dorsolateral prefrontal cortex | 46 | L | -39 | 32 | 19 | 8.29 |
| Fusiform gyrus/Inferior temporal gyrus | 37 | L | -48 | -43 | -15 | 8.45 |
| Premotor cortex | 6 | L | -45 | 2 | 37 | 7.87 |


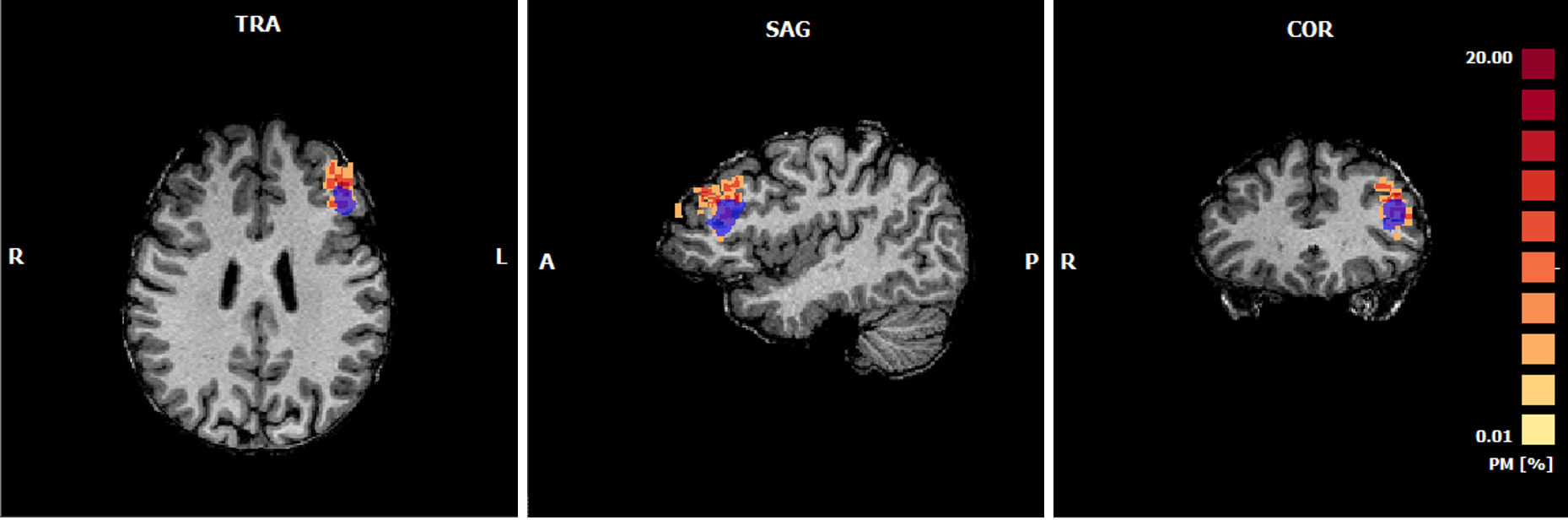


**Fig. 1.** Overlap between the probabilistic maps of the ROIs selected online during the functional localizer (across all subjects) (Talairach center of gravity mean coordinates: -39.18 ± 2.93, 35.09 ± 7.66, 27.85 ± 7.53; Number of voxels: 393) and the ROI on the left Dorsolateral Prefrontal Cortex (left-DLPFC) selected based on the whole-brain RFX analysis of the localizer task (Talairach cluster peak voxel center coordinates: -39, 32, 19; Number of voxels: 1626). Both ROIs were selected based on the contrast Pain Images > Neutral Images. The percentage of subjects in which each voxel was selected is represented accordingly to the color range from yellow to red. The offline ROI is represented in blue.

**Table 3.** ROI-GLM analysis of the three imagery tasks (‘Self’: imagine itself in painful situations; ‘Other’: imagine a loved-one in painful situations; ‘Down-regulation’: imagine pleasure/pain-relief situations) during the neuromodulation runs with (NF1, NF2, and NF3) and without (train and transfer) feedback. Mean beta values for contrasts of interest with respective standard error (SE) and degrees of freedom (df), and t-statistics (t-values) with statistical significance (p-values).

|  | | ***df*** | ***Mean beta*** | ***SE*** | ***t*** | ***p*** |
| --- | --- | --- | --- | --- | --- | --- |
| ***Train*** | *Self > Down-regulation*  *Other > Down-regulation*  *Self > Other* | 16  16  16 | 0.229  0.234  -0.005 | 0.088  0.066  0.061 | 2.597  3.557  -0.087 | 0.019447  0.002627  0.931712 |
| ***NF1*** | *Self > Down-regulation*  *Other > Down-regulation*  *Self > Other* | 16  16  16 | 0.295  0.291  0.004 | 0.079  0.120  0.081 | 3.737  2.415  0.051 | 0.001797  0.028066  0.960066 |
| ***NF2*** | *Self > Down-regulation*  *Other > Down-regulation*  *Self > Other* | 16  16  16 | 0.240  0.276  -0.037 | 0.106  0.082  0.060 | 2.257  3.357  -0.615 | 0.038331  0.004008  0.547382 |
| ***NF3*** | *Self > Down-regulation*  *Other > Down-regulation*  *Self > Other* | 16  16  16 | 0.328  0.227  0.101 | 0.111  0.054  0.079 | 2.956  4.167  1.280 | 0.009294  0.000727  0.218888 |
| ***NF1+NF2+NF3*** | *Self > Down-regulation*  *Other > Down-regulation*  *Self > Other* | 16  16  16 | 0.287  0.256  0.023 | 0.080  0.073  0.025 | 3.579  3.614  0.919 | 0.002509  0.002328  0.371978 |
| ***Transfer*** | *Self > Down-regulation*  *Other > Down-regulation*  *Self > Other* | 16  16  16 | 0.424  0.335  0.089 | 0.127  0.099  0.061 | 3.345  3.376  1.470 | 0.004112  0.003852  0.16095 |

**Table 4.** Clusters of significant signal change in the right (R) and left (L) hemispheres, from anterior to posterior coordinates, when participants were imagining painful situations from the ‘Self’ perspective vs. down-regulation (q(FDR) = 0.03) (X, Y, and Z represent the cluster peaks in Talairach coordinates).

| ***Region*** | ***Brodmann area*** | ***L/R*** | ***Cluster peak coordinates*** | | | ***t-Score*** | ***Nr. Voxels*** |
| --- | --- | --- | --- | --- | --- | --- | --- |
|  |  |  | ***X*** | ***Y*** | ***Z*** |  |  |
| *Insula* | *13* | *R* | *30* | *17* | *7* | *6.044* | *651* |
| *Thalamus* | *50* | *R* | *6* | *-22* | *7* | *5.580* | *291* |
| *Thalamus* | *50* | *L* | *-3* | *-4* | *10* | *7.244* | *573* |
| *Insula* | *45* | *L* | *-30* | *14* | *10* | *7.053* | *971* |
| *Dorsal striatum* |  | *L* | *-18* | *2* | *10* | *7.651* | *955* |
| *Dorsal striatum* |  | *R* | *12* | *2* | *10* | *5.371* | *502* |
| *Dorsolateral prefrontal cortex* | *46* | *L* | *-42* | *35* | *19* | *5.564* | *286* |
| *Somatosensory cortex* | *40* | *L* | *-42* | *-28* | *34* | *5.447* | *742* |
| *Anterior cingulate cortex* | *32* | *L* | *-9* | *17* | *34* | *5.820* | *440* |
| *Anterior cingulate cortex* | *8* | *R* | *9* | *11* | *37* | *5.792* | *574* |
| *Premotor cortex* | *6* | *R* | *33* | *-10* | *49* | *5.363* | *935* |
| *Intraparietal sulcus* | *39* | *L* | *-39* | *-52* | *52* | *5.028* | *769* |
| *Premotor cortex* | *6* | *L* | *-30* | *-10* | *55* | *5.114* | *540* |
| *Intraparietal sulcus* | *7* | *L* | *-15* | *-64* | *58* | *6.946* | *821* |

**Table 5.** Clusters of significant signal change in the right (R) and left (L) hemispheres, from anterior to posterior coordinates, when participants were imagining painful situations from the ‘Other’ perspective vs. down-regulation (q(FDR) = 0.03) (X, Y, and Z represent the cluster peaks in Talairach coordinates).

| ***Region*** | ***Brodmann area*** | ***L/R*** | ***Cluster peak coordinates*** | | | ***t-Score*** | ***Nr. Voxels*** |
| --- | --- | --- | --- | --- | --- | --- | --- |
|  |  |  | ***X*** | ***Y*** | ***Z*** |  |  |
| *Insula* | *13* | *L* | *-30* | *17* | *4* | *7.764* | *901* |
| *Thalamus* | *50* | *R* | *18* | *-19* | *10* | *6.035* | *591* |
| *Dorsal striatum* |  | *R* | *15* | *8* | *13* | *6.951* | *799* |
| *Dorsal striatum* |  | *L* | *-15* | *1* | *14* | *6.910* | *934* |
| *Thalamus* | *50* | *L* | *-12* | *-19* | *14* | *6.213* | *949* |
| *Supramarginal gyrus (temporoparietal junction)* | *39* | *L* | *-45* | *-43* | *28* | *5.414* | *429* |
| *Supramarginal gyrus (temporoparietal junction)* | *40* | *L* | *-51* | *-28* | *31* | *4.516* | *932* |
| *Precuneus* | *7* | *L* | *-3* | *-64* | *34* | *4.598* | *60* |
| *Dorsolateral prefrontal cortex* | *8* | *L* | *-33* | *20* | *37* | *5.133* | *327* |
| *Anterior cingulate cortex* | *32* | *L* | *-9* | *11* | *37* | *7.211* | *515* |
| *Anterior cingulate cortex* | *32* | *R* | *12* | *14* | *37* | *4.968* | *598* |
| *Premotor cortex* | *6* | *L* | *-39* | *-4* | *37* | *5.255* | *717* |
| *Premotor cortex* | *6* | *L* | *-24* | *2* | *43* | *7.173* | *608* |
